# Social media use as a source of information by acne vulgaris patients

**DOI:** 10.1111/jocd.16477

**Published:** 2024-07-17

**Authors:** Bahar Ürün Ünal, Abdullah Demirbaş, Burcu Gök Erdoğan

**Affiliations:** ^1^ Department of Family Medicine, Faculty of Medicine Selcuk University Konya Turkey; ^2^ Department of Dermatology, Faculty of Medicine Kocaeli University Kocaeli Turkey

**Keywords:** acne vulgaris, cosmetics, social media, treatment

## Abstract

**Background:**

Social media and internet usage is undeniably high. Misinformation obtained from the internet and wrong treatment methods can cause serious problems in patients with acne vulgaris (AV). In this study, the sociodemographic data of AV patients, their frequency of using the internet as an information source, the relationship between them, and their attitudes and behaviors regarding their disease due to these programs were examined.

**Methods:**

481 patients aged 14 and over diagnosed with AV were included in the study. It was conducted in a descriptive cross‐sectional type. Acne severity of all patients included in the study was determined using the Global Acne Grading System.

**Results:**

78.3 percent of participants use social media to get information about AV. It was determined that men and single people used social media about their illnesses at a statistically significantly higher rate than women and married people (*p* = 0.004). In addition, patients aged 13–18 and high school graduates use social media as a source of information about their diseases, and this rate is statistically significantly higher (*p* < 0.001).

**Conclusion:**

Especially in the last decade, the use of social media tools to spread health messages has increased significantly. Because it has a chronic course and can cause cosmetic problems, AV patients may frequently resort to communication sources such as social media. Considering the possibility of social media misinforming patients, physicians should be aware that their patients with AV frequently use social media and should improve themselves in creating correct awareness on this issue.

**Abbreviations**
AV: Acne Vulgaris
CAM: Complementary and Alternative Medicine
GAGS: Global Acne Grading System

## 1 INTRODUCTION

Social media is the most widely used internet‐based communication tool and has an important place in our lives in the field of health.^1^ Facebook, YouTube, WhatsApp, and Instagram are the most frequently preferred social media platforms in this field.^2^ The most frequently used search engine is Google.^3^ People try to diagnose their diseases through social media, learn about their diseases, and look for alternative treatments.

Acne vulgaris (AV) is a chronic inflammatory disease of the pilosebaceous unit that usually affects the face and upper body and is accompanied by seborrhea, comedones, erythematous papules, pustules, deep or pseudocystic nodules, and sometimes scars.^4^, ^5^ AV has the highest prevalence, especially in adolescents, and affects approximately 9.4% of the world population.^6^ Although the burden of AV‐related disease has a global distribution, the prevalence of this burden in the population continues to increase over time.^7^ Acne can last for years and leave permanent scars on the skin despite treatment.^8^ AV can have significant psychological and social consequences for individuals dealing with it. For this reason, it was stated that 82.4% of the patients who applied to the dermatology outpatient clinic used the internet and 65.4% used social media to obtain information about their diseases.^9^ Patients with AV may frequently resort to communication sources such as social media because the disease has a chronic course, may cause cosmetic problems, and negatively affects the patients' psychosocial functionality and quality of life.^10^

In this study, it was aimed to examine the frequency of using the internet and social media in cases diagnosed with AV, which platforms are used more frequently, the reasons for applying to these platforms, and the factors affecting the application, as well as to better understand the potential impact of social media on patients with AV and to determine communication tendencies.

## 2 MATERIALS AND METHODS

### 2.1 Study Design and Ethical Approval

This descriptive cross‐sectional study was conducted at a training and research hospital in Turkey between February and April 2021.

The aims and procedures of the study were evaluated and approved by our ethics committee before performing the relevant analyzes (Date: September 28, 2021; Decision no: 2021/430). It was confirmed that all aims and instruments of the study were designed in accordance with the ethical standards of the institutional research committee and the declaration of Helsinki and its later amendments.

481 (94.3%) of 510 patients aged 14 and over who applied to the Dermatology Polyclinic between February and April 2021 and were diagnosed with AV were included in the study. 19 patients who refused to participate in the study were not included in the study, and relevant data were not collected. Written informed consent was obtained from all participants before participating in the project. It was planned to include all patients with AV who volunteered and signed informed consent during data collection, so a targeted sample size or group could not be created.

### 2.2 Data Collection

Participants were asked to complete a 4‐part, anonymous, self‐administered survey. In the first part, there are sociodemographic questions including the participants' age, gender, educational status, and marital status. In the second part, questions were asked to evaluate how long the disease had been going on, the medications used for AV treatment, the presence of AV in the family, and the location of the acne. In the third part, questions were asked aiming to question the participants' social media and internet usage habits and attitudes for AV. Participants were asked questions about the social media platforms they use most frequently, what information they search on the internet, whether they share this information with their doctors, and whether they follow TV/radio programs. In the fourth section, participants were asked about the foods they thought aggravated their acne.

Acne severity was determined by the Global Acne Grading System (GAGS) of all patients included in the study. GAGS, developed by Doshi et al., is used to calculate acne severity. GAGS is a measurement system that scores acne severity according to the type of lesion and the distribution of acne in the body. According to this system, acne severity is calculated by multiplying the score of the most severe lesion (no lesion = 0, comedone = 1, papule = 2, pustule = 3, nodule = 4) detected from six determined regions (face, chest, and upper back) by a constant factor. According to the GAGS between 0 and 44; 0 points: no acne, 1–18 points: mild, 19–30 points: moderate, 31–38 points: severe, 39 points and above: very severe.

### 2.3 Statistical Analysis

IBM SPSS for Windows, Version 25.0 (IBM Corp., Armonk, NY, USA) was used for all analyzes conducted with a significance threshold of *p* < 0.05. The normal distribution of variables was evaluated by examining histogram graphs. Descriptive statistics include the mean ± standard deviation for normally distributed continuous variables. Intergroup analyzes for categorical variables were performed with the chi‐square test.

## 3 RESULTS

481 patients with AV were included in the study. 60.1% of the patients were female and 39.9% were male. 42.4% were between the ages of 13–18, 43.9% were between the ages of 19–25, and 13.7% were aged 26 and over. The mean age was 19.7 ± 4.12 years. 73.6% of the participants were single and 26.4% were married. 42.6% were primary school graduates, 37.2% were high school graduates, and 20.2% were university graduates. 52.2% of the patients were students, 10.2% were housewives, 18.1% were workers, 17.3% were civil servants, and 2.3% were not working. 33.3% of the participants were always smokers, 47.6% were never smokers, 14.6% were occasional smokers, and 4.6% had quit smoking. 3% of the patients used alcohol constantly, 94% did not use alcohol at all, and 3% used alcohol occasionally. When the participants were questioned about their use of social media as a source of information about their diseases, it was found that 24.3% of the patients used social media, 21.4% did not use it, and 54.3% sometimes used social media.

24.3% of the participants did not use the internet or social media regularly, 54.3% sometimes, and 21.4% never used the internet or social media. Social media and internet use regarding AV was statistically significantly higher in men, participants between the ages of 13–18, single people, those with facial acne only, high school graduates, those without a family history of acne, and those whose disease duration was within the first 2 years. However, no statistically significant relationship was found between acne severity and acne severity (Table 1).

**TABLE 1:** Characteristics of the respondents.

Patient characteristics	Social media and internet use for AV								
	Yes		No		Sometimes		Overall		
	*n*	%	*n*	%	*n*	%	*n*	%	*p*
Gender									
Female	73	25.3	75	26.0	141	48.8	289	60.1	**0.004**
Male	44	22.9	28	14.6	120	62.5	192	39.9	
Age									
13–18 years old	108	52.9	28	13.7	68	33.3	204	42.4	**0.000**
19–25 years old	3	1.4	62	29.4	146	69.2	211	43.9	
26 years and above	6	9.1	13	19.7	47	71.2	66	13.7	
Marital status									
Married	6	4.7	39	30.7	82	64.6	127	26.4	**0.000**
Single	111	31.3	64	18.1	179	50.6	354	73.6	
Disease severity									
1	63	24.5	65	25.3	129	50.2	257	53.4	0.010
2	49	26.1	27	14.4	112	59.6	188	39.1	
3	5	29.4	3	17.6	9	52.9	17	3.5	
4	0	0.0	8	42.1	11	57.9	19	4.0	
Acne localization									
Face	80	23.9	55	16.4	200	59.7	335	69.6	**0.000**
Body	9	30.0	14	46.7	7	23.3	30	6.2	
Face and body	28	24.1	34	29.3	54	46.6	116	24.1	
Education level									
Primary education	96	46.8	48	23.4	61	29.8	205	42.6	**0.000**
High school	15	8.4	22	12.3	142	79.3	179	37.2	
University	6	6.2	33	34.0	58	59.8	97	20.2	
Family history of AV									
Absent	45	45.5	16	16.2	38	38.4	99	20.6	**0.000**
Present	72	18.8	87	22.8	223	58.4	382	79.4	
Disease duration									
In the first 2 years	88	50.3	30	17.1	57	32.6	175	36.4	**0.000**
Within 2–4 Years	23	15.4	30	20.1	96	64.4	149	31.0	
Within 4–6 Years	6	3.8	43	27.4	108	68.8	157	32.6	
Age (n)	19.7 ± 4.12 (min‐max: 13–42)								

*Note:* Bold values indicate *p* < 0.05.

As indicated in Table 2, 61.5% of AV patients were using only topical treatment, 5.4% were using isotretinoin, and 33.1% were using both antibiotics and topical treatment. 79.4% of the patients had received any acne treatment before. According to the GAGS, 53.4% of the patients were mildly severe, 39.1% were moderately severe, 3.5% were severe, and 4% were very severe. 79.4% of the patients had a family history of acne.

**TABLE 2:** Disease characteristics of AV patients.

Patient characteristics	*n*	%
Acne treatment method		
Topical Only	296	61.5
Isotretinoin	26	5.4
Antibiotics and topical	159	33.1
Previously used acne treatment		
Yes	365	79.4
No	116	20.6
GAGS		
Mild Severity	257	53.4
Moderately Severe	188	39.1
Severe	17	3.5
Very severe	19	4.0
Presence of AV in the family		
Yes	382	79.4
No	99	20.6

76.4% of AV patients using social media use YouTube, 71.2% use Google, 59.5% use Twitter (X), 59% use Facebook, 59% use Instagram, and 11.6% use social media. used patient blogs (Figure 1).

**FIGURE 1:**
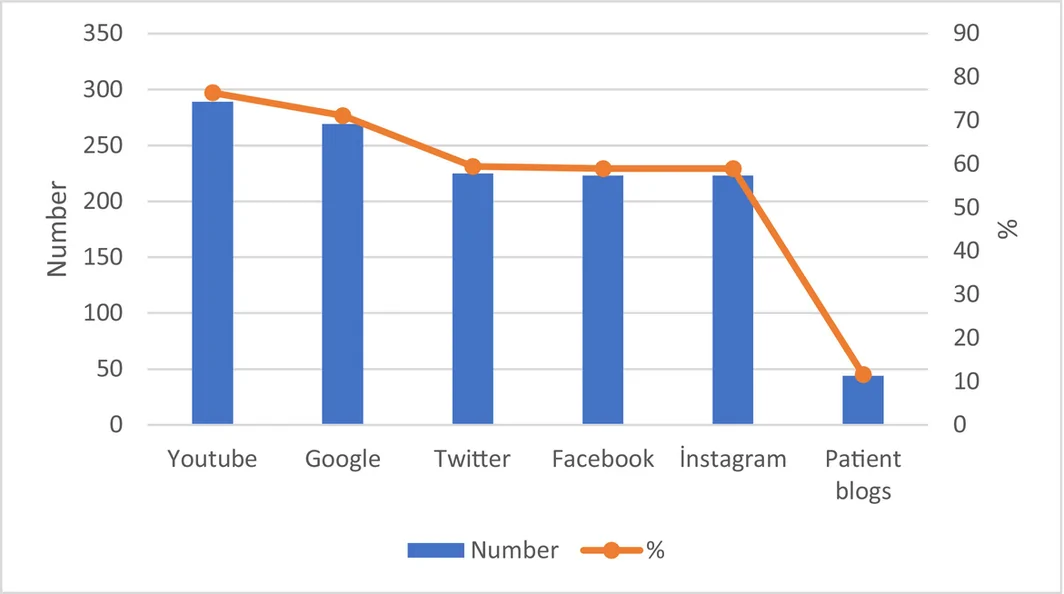
Internet and social media platforms most frequently used by patients for AV inquiries (The respondents were allowed to choose more than one option).

90.2% of the patients were searching for doctors, 77.2% for medications, 96.3% for acne‐related information, and 96.8% for complementary and alternative medicine (CAM) on the internet. 41.3% of AV patients using social media openly shared what they saw on social media with their doctor, 57.1% implicitly shared it with their doctor, and 1.6% did not share what they saw on social media with their doctor at all. AV patients using social media were asked, “If you asked a question to any doctor by sending a photo via social media, would you trust the answers?” 15.1% of the patients answered this question as yes, 15.3% as no, and 69.6% as if they would still go for examination (Table 3).

**TABLE 3:** Characteristics of social media use of AV patients using social media.

Characteristics	*n*	%
What information do you search online?^a^		
Doctors	341	90.2
Medicines	292	77.2
Information about acne	364	96.3
CAM	366	96.8
Would you share what you see on social media with your doctor?		
Yes, clearly	156	41.3
Yes, implicitly	216	57.1
No	6	1.6
If you sent a photo to any doctor on social media and asked a question, would you trust the answers?		
Yes	57	15.1
No	58	15.3
I trust but I still go to get examined	263	69.6
Do you try to reach your doctor via social media?		
Yes	45	11.9
No	282	74.6
Sometimes	51	13.5

^a^ More than one option is marked.

27.2% of AV patients stated that they follow TV/radio programs. Of the patients who follow TV/radio programs, 48.1% stated that they researched what they saw on the internet, 15.3% stated that they asked their doctor about what they saw, 19.8% asked other patients about what they saw, and 16.8% stated that they researched what they saw appropriate. 83.2% of the patients who followed TV/radio programs stated that they applied what they saw in the program to their acne. 27.5% of these patients stated that they openly asked their doctor about what they saw in the program, 61.8% stated that they asked their doctor implicitly, and 10.7% stated that they did not share this issue with their doctor (Table 4).

**TABLE 4:** Attitudes and behaviors of patients who follow TV/Radio programs about their diseases (*n* = 131).

	*n*	%
Do you follow TV/radio programs? (*n* = 481)		
Yes	131	27.2
No	350	72.8
Would you research what you see in the program?		
Yes, I'm searching online	63	48.1
Yes, I ask my doctor	20	15.3
Yes, I ask other patients	26	19.8
I investigate if I deem it appropriate.	22	16.8
Would you apply what you see in the program to your acne?		
Yes	109	83.2
No	22	16.8
Would you share what you saw in the program with your doctor?		
Yes, clearly	36	27.5
Yes, implicitly	81	61.8
No	14	10.7

The patients were questioned about the relationship between their acne and diet. 87% of patients The patients were questioned about the relationship between their acne and diet. 87% of patients stated that some foods negatively affected their acne. According to patient opinions, the foods that most negatively affect acne are listed as follows: fatty foods (55.1%), fried products (45.9%), cola (45.7%), chocolate (42.2%), ketchup (39.9%), packaged foods (39.7%), seeds (34.3%), nuts (31%). Hot and spicy foods and alcohol are in lower amounts.

## 4 DISCUSSION

Recently, social media has become a popular source of health information for all individuals. Patients may be influenced by the information they obtain on social media and the internet and use unsafe medications, and try different treatment models without consulting a doctor.^11^, ^12^

In the study, 24.3% of patients with AV use social media platforms constantly, and 54.3% sometimes use social media platforms for acne treatment. This high rate of social media use has also been detected in previous studies reporting the use of social media for acne treatment.^13^, ^14^ In another study conducted in Thailand, when patients were asked where they obtained information about AV, the first answer was “from friends” and the second was “social media.”^15^

In terms of gender, male patients with AV were more sensitive to the acne treatment recommendations they received from social media (*p* = 0.004). In many studies in the literature, women's social media usage was statistically higher than men.^13^, ^14^, ^16^ We think that the reason for this situation, which is contrary to the literature in our study, may be that there are differences in endocrine levels between genders, that is, the prevalence of acne is higher in males during adolescence.^17^ Many studies have reported that the prevalence and severity of acne are significantly higher in adolescent males.^18^ The prevalence of acne among Europeans aged 15–24 is 58.28% in men and 56.97% in women.^19^ In an epidemiological study on acne among undergraduate students in North East China, acne among adolescents was found to be 52.74% in men and 49.65% in women.^17^ Similarly, a study conducted in Singapore showed that more men than women suffered from teenage acne (61.3% vs. 38.8%).^20^ Similarly, many studies have shown that men have more acne problems than women during adolescence.^21^, ^22^ Since the majority of the participants are young people and individuals in adolescence, we think that men use social media related to AV more. Likewise, the reason why single AV patients use AV‐related social media at a high rate (81.9%) may be that most of the participants are young individuals. In a similar study, the majority of patients (76.3%) were single.^23^ Another reason may be that single individuals spend more time on social media. In a study conducted in Turkey, it was seen that single people spent more time on social media than married people.^24^

When social media usage status was examined according to the duration of the disease, it was seen that AV patients most frequently used social media within the first 2 years of the disease. This may be due to the fact that, especially in adolescence and young individuals, they attach more importance to appearance and want to be liked, so they want to find a faster solution to a dermatological problem and seek treatment quickly. Likewise, we think that the reason why the rate of using social media is higher in patients with acne localization on the face is due to the same reason in young individuals, and that it creates more anxiety in patients because the face is a visible area.

Some studies have shown that acne treatment information on Instagram is unreliable.^11^ In our study, it was seen that the participants used YouTube most frequently among the social media platforms, followed by Google, Twitter (X), Facebook and Instagram, respectively. We think that this is because AV is more common in the young population and YouTube is very popular among young people. Similar studies have also found that the most frequently used social media platforms are the platforms most preferred by the young population.^14^, ^16^

Social media has a huge impact on acne treatment, especially among teenagers and young adults. This study shows that patients with AV frequently resort to the internet and social media to seek treatment for their disease and to obtain information about their disease. However, in addition to providing quick access to information, social media is a platform where not only physicians but also nonspecialists in the field of health share posts about diseases. According to Borba et al.'s^25^ study, the acne education videos that patients searched on the internet were clearly inaccurate and of poor quality, and incorrect or irregular treatments recommended on social media could contribute to the exacerbation of acne. For this reason, patients easily access unreliable and inaccurate information and can try different forms of treatment without a doctor's control. There is a lot of misinformation on social media regarding acne treatment, highlighting the importance of appropriate patient education for those suffering from acne. In addition, these findings should warn healthcare professionals because patients trying treatments that are not suitable for them, are not included in the guidelines, and have serious side effects on the skin may harm individuals.

In our study, it was determined that patients mostly consulted social media regarding CAM regarding AV. In a similar study conducted by Yousaf et al.,^13^ it was observed that patients mostly turned to social media for nonprescription products. This suggests that patients generally use social media to seek solutions to their diseases. In another study conducted in Turkey, it was observed that patients most frequently turned to social media to get general information about their diseases.^14^

Our study is one of the limited number of studies investigating the internet and social media usage habits of patients with AV, their views on health programs in traditional media (TV/radio), and how much of the information they encounter in the media they share with their doctors. As a result, access to social media and the internet is becoming easier day by day and individuals are seeking treatment for their diseases on these platforms; It shows that physicians should use social media and the internet more actively to share accurate information about AV and other diseases, to provide more information to patients, and to prevent the sharing of misinformation.

### 4.1 Limitations

Some possible limitations of the research are as follows. Since it is a single‐center study and the study was conducted in the city center, access to social media and the internet may differ in rural areas, the generalizability of the results to AV patients nationwide or globally is limited. Since the prevalence of AV is higher in young age and adolescence, most of the patients represented the young population and were still in education. Therefore, the education level of the patients was relatively low. Additionally, patients' knowledge about AV treatments and their side effects was not measured. Therefore, it is necessary to investigate more comprehensively whether AV patients are aware of the accuracy or inaccuracy of the information they obtain from social media and the internet and whether they have information about the side effects of these treatment methods.

## 5 CONCLUSIONS

Our data show that 78.6% of AV patients consult social media and the internet about their illness, this is more common in men than women, and is higher in single people, those in the first 2 years of the disease, and the young population and adolescents. Due to the presence of a lot of incorrect information on the internet and social media about AV disease and treatment methods, which may lead to dangerous consequences, especially on the skin; Patients should be informed about the dangers of social media, they should be informed about the harms caused by drugs used without a prescription and without a doctor's recommendation, and physicians should be encouraged to use social media and the internet more actively. We hope that our study will raise awareness on the subject for physicians.

## AUTHOR CONTRIBUTIONS

All authors have reviewed and approved the article for submission. Conceptualization: Bahar Urun Unal, Data curation: Bahar Urun Unal, Abdullah Demirbas, Formal analysis: Bahar Urun Unal, Burcu Gok Erdogan, Funding acquisition: Bahar Urun Unal, Investigation: Bahar Urun Unal, Burcu Gok Erdogan, Methodology: Bahar Urun Unal, Abdullah Demirbas, Software: Bahar Urun Unal, Burcu Gok Erdogan, Project administration: Bahar Urun Unal, Burcu Gok Erdogan, Resources: Bahar Urun Unal, Burcu Gok Erdogan, Supervision: Bahar Urun Unal, Burcu Gok Erdogan, Validation: Bahar Urun Unal, Abdullah Demirbas, Visualization: Bahar Urun Unal, Abdullah Demirbas, Writing–original draft: Bahar Urun Unal, Burcu Gok Erdogan, Writing–review & editing: Bahar Urun Unal, Burcu Gok Erdogan.

## FUNDING INFORMATION

None.

## CONFLICT OF INTEREST STATEMENT

Authors declare that they have no conflict of interest.

## ETHICS STATEMENT

The aims and procedures of the study were evaluated and approved by our ethics committee before performing the relevant analyzes (Date: September 28, 2021; Decision no: 2021/430). It was confirmed that all aims and instruments of the study were designed in accordance with the ethical standards of the institutional research committee and the declaration of Helsinki and its later amendments.

## Data Availability

Data supporting the findings of this study are available from the corresponding author upon reasonable request.
