# Correction to “Social Media Use as a Source of Information by Acne Vulgaris Patients”

**DOI:** 10.1111/jocd.70130

**Published:** 2025-03-20

Cite: B. Ü. Ünal, A. Demirbaş, B. G. Erdoğan, “Social Media Use as a Source of Information by Acne Vulgaris Patients,” *Journal of Cosmetic Dermatology* 23, no. 10 (2024): 3312–3318, https://doi.org/10.1111/jocd.16477.

In paragraph first of the “Materials and Methods/Study Design and Ethical Approval” section, the text “This descriptive cross‐sectional study was conducted at a training and research hospital in Turkey between February and April 2021.” was incorrect. This should have read: “This descriptive cross‐sectional study was conducted at a training and research hospital in Turkey between February and April 2022.”

In the third paragraph of the “Materials and Methods/Study Design and Ethical Approval” section, the text “481 (94.3%) of 510 patients aged 14 and over who applied to the Dermatology Polyclinic between February and April 2021 and were diagnosed with AV were included in the study.” was incorrect. Instead, it should have been written as follows: “481 (94.3%) of 510 patients aged 14 and over who applied to the Dermatology Polyclinic between February and April 2022 and were diagnosed with AV were included in the study.”

We apologize for this error.

